# *Spartina alterniflora* Ecosystem Stability: Insights Into the Interplay Between Soil Bacteria and Their Functional Traits

**DOI:** 10.1002/ece3.71096

**Published:** 2025-04-03

**Authors:** Xue Mo, Zhenming Zhang, Yinglong Chen, Shijun Zhou, Yi Li, Siqi Zhao, Shiqiang Zhao, Xuanming Chen, Bo Wu, Mingxiang Zhang

**Affiliations:** ^1^ School of Ecology and Nature Conservation Beijing Forestry University Beijing China; ^2^ School of Agriculture and Environment, and UWA Institute of Agriculture University of Western Australia Perth Western Australia Australia; ^3^ Beijing Top Green Ecological Technology Limited Company Beijing China

**Keywords:** bacterial diversity, bacteriome stability, co‐occurrence network, core and specialized function, keystone taxa, *Spartina alterniflora*

## Abstract

The relationship between soil microbiome stability and diversity remains a topic of debate. Our study aims to investigate the relationship between soil microbiome stability and diversity in different wetland types invaded by *Spartina alterniflora* and to reveal the mechanisms driving functional influences on this relationship during the later‐stage development of the *S. alterniflora* invasion system. To investigated the structure, diversity, and functional traits of soil bacteria associated with *S. alterniflora* and their impact on bacteriome stability we conducted 16S rRNA sequencing of soils from two types of wetlands dominated by the invasive plant *S. alterniflora* at different growth stages, situated in temperate (salt marsh wetland) and subtropical (mangrove wetland) regions, and assessed bacteriome stability and its driving factors. Subsequently, we analyzed environmental and bacterial changes between the two sites and constructed co‐occurrence networks among taxonomic groups and functional traits. The differences in the late‐stage development of the two *S. alterniflora* ‐invaded wetland systems suggest that bacterial communities with higher diversity tend to exhibit greater stability. Keystone genera play both direct and indirect roles in regulating bacteriome stability, and all belong to dominant phyla. Furthermore, biological factors significantly outweigh nonbiological factors in driving stability. In contrast, core functions (broad functions) and specialized functions such as “nitrogen metabolism” and “sulfur metabolism” decrease bacteriome stability. Their enhancement of these metabolic processes correlates with reduced community stability, which is the key to the differences observed in the two invaded systems. This study advances our understanding of the relationship between soil microbial diversity and ecosystem stability, highlighting the importance of keystone taxa and functional traits for soil microbiome stability. It enhances our ability to predict microbial community transitions. It enhances a scientific basis for the management of *S. alterniflora* invasion.

## Graphical abstract

In the late‐stage development of the two *S. alterniflora* ‐invaded wetland systems, bacterial communities with higher diversity tend to be more stable, and the environmental factors are not the primary driver of stability. Keystone genera play both direct and indirect roles in regulating bacteriome stability. The increased metabolic processes of core functions and specialized functions were key factors in reducing community stability between the two types of wetland systems.

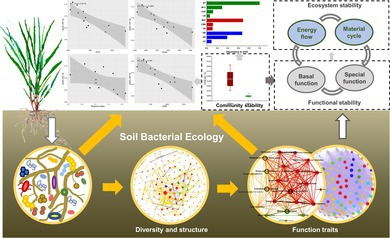

## 1 Introduction

Invasive species pose a severe threat to biodiversity and stability in native ecosystems worldwide (Marco et al. 2011). *Spartina alterniflora* , a C4 plant ranked among the world's hundred most dangerous alien invasive species, is native to the Atlantic and Gulf coasts of North America, was intentionally introduced to China in 1979 for coastal erosion control (Yuan et al. 2019), due to its high‐quality aboveground biomass and litter (Perez 2015), but ultimately causes extensive damage to coastal tidal wetlands in China. Its invasion leads to the occupation of native plant ecological niches, reducing plant diversity and subsequently diminishing the variety of root exudates entering the soil, resulting in lower microbial nutrient source diversity and a decline in microbial diversity. This loss of soil microbial diversity poses a significant threat to ecosystem stability.

Soil microbial activity is a vital driver of material cycling and nutrient transformation in soil ecosystems, and the stability of microbial communities plays a significant role in maintaining soil health, ecosystem services, plant growth, ecological balance, and resilience (Liu et al. 2022a; Lurgi et al. 2019; Philippot et al. 2021). The loss of soil microbial diversity is a globally recognized threat to ecosystems, leading to research focusing on the relationship between microbial diversity, community stability, and ecosystem functioning (Wu et al. 2022; Zhou et al. 2020). Efforts have been directed towards developing ecological models and analyzing simple microbial communities with well‐defined genetic backgrounds (Bucci et al. 2012; Schluter and Foster 2012). However, to date, there has been little application of relevant research to the management of invasive plants. This highlights the potential of manipulating soil microbial community stability as a means to regulate the stability of invasive ecosystems, which could be a crucial approach for future invasive species management. This could be the key to managing invasive species by regulating the stability of invasive ecosystems through the intervention of soil microbial community stability.

Increasing evidence suggests that microbiome stability primarily stems from species diversity. Increased microbial diversity and abundance promote functional diversity, resulting in a more stable microbial community. Conversely, species loss typically results in impaired ecosystem functioning (Liu et al. 2022b; Zheng et al. 2022). Variations in microbial species diversity can significantly impact the balance of metabolic functions and reduce functional redundancy (Huang et al. 2021). Furthermore, functional imbalance may lead to the collapse of microbial interaction networks, resulting in decreased community stability (Louca et al. 2018). Studies have shown that changes in influencing factors such as environmental conditions and interactions among microbial taxa can affect microbial networks and community stability by regulating keystone taxa (Banerjee et al. 2018; Liu et al. 2022a). Networks with a higher number of key taxonomic groups are generally more stable. The removal of these keystone taxa can lead to network instability, causing disturbances to ecosystem functioning (Banerjee et al. 2018). Similarly, the key functions of microorganisms significantly influence the structural stability of microbial communities (Xun et al. 2021). Methods investigating the effects of microbial diversity and functional traits on stability often involve the removal of putative keystone taxa, followed by gradual dilution to assess changes in community diversity and functional traits, and further analysis of the mechanisms underlying their effects on microbiome stability (Xun et al. 2019; Xun et al. 2021). Additionally, microbial co‐occurrence networks have proven to be particularly valuable in investigating complex relationships among numerous microbial species (Berry and Widder 2014; Perez 2015). Network metrics such as average degree and closeness centrality can be utilized to statistically identify keystone taxa and metabolic functions (Banerjee et al. 2018; Ma et al. 2020a).

Xun et al. (2021) conducted a co‐occurrence network analysis on previously targeted 16S rRNA gene and shotgun metagenomic sequencing data. They identified keystone taxa and associated functional features, finding that specialized metabolic functions related to nitrogen and phosphorus metabolism of these keystone taxa were crucial in maintaining the stability of the soil microbial community. These key functions may include additional keystone taxa and functions related to maintaining soil microbial stability. Moreover, certain key “broad functions” may also play a crucial role in shaping the structure of soil microbial communities (Huang et al. 2021). This is because the formation of “broad function” communities is associated with functional redundancy; for example, multiple bacterial taxa can participate in carbon and nitrogen cycles. Therefore, even if certain taxa are impacted by environmental stress, other taxa can “take over” these essential functions, ensuring that the overall functional structure does not collapse (Kembel et al. 2010). However, it remains unclear whether these key “specialized functions” and “broad functions” of bacteria, relative to environmental factors, play a decisive role in bacteriome stability and their impact mechanisms.

Therefore, in this study, we focused on identifying the main drivers of bacteriome stability in the later‐stage development of the *S. alterniflora* invasion system and hypothesized that structural stability is primarily driven by key bacterial taxa, with both broad and specialized functions, playing a facilitative role, rather than environmental factors. To test these hypotheses, we analyzed the keystone taxa and metabolic functions driving bacterial community stability and validated the driving mechanism of community diversity on stability. We further analyzed the contributions to stability in terms of key communities and core functions, thereby exploring the potential of regulating microbial diversity through intervention in driving factors to manage the invasion of *S. alterniflora* .

## 2 Material and Methods

### 2.1 Study Area

**FIGURE 1:**
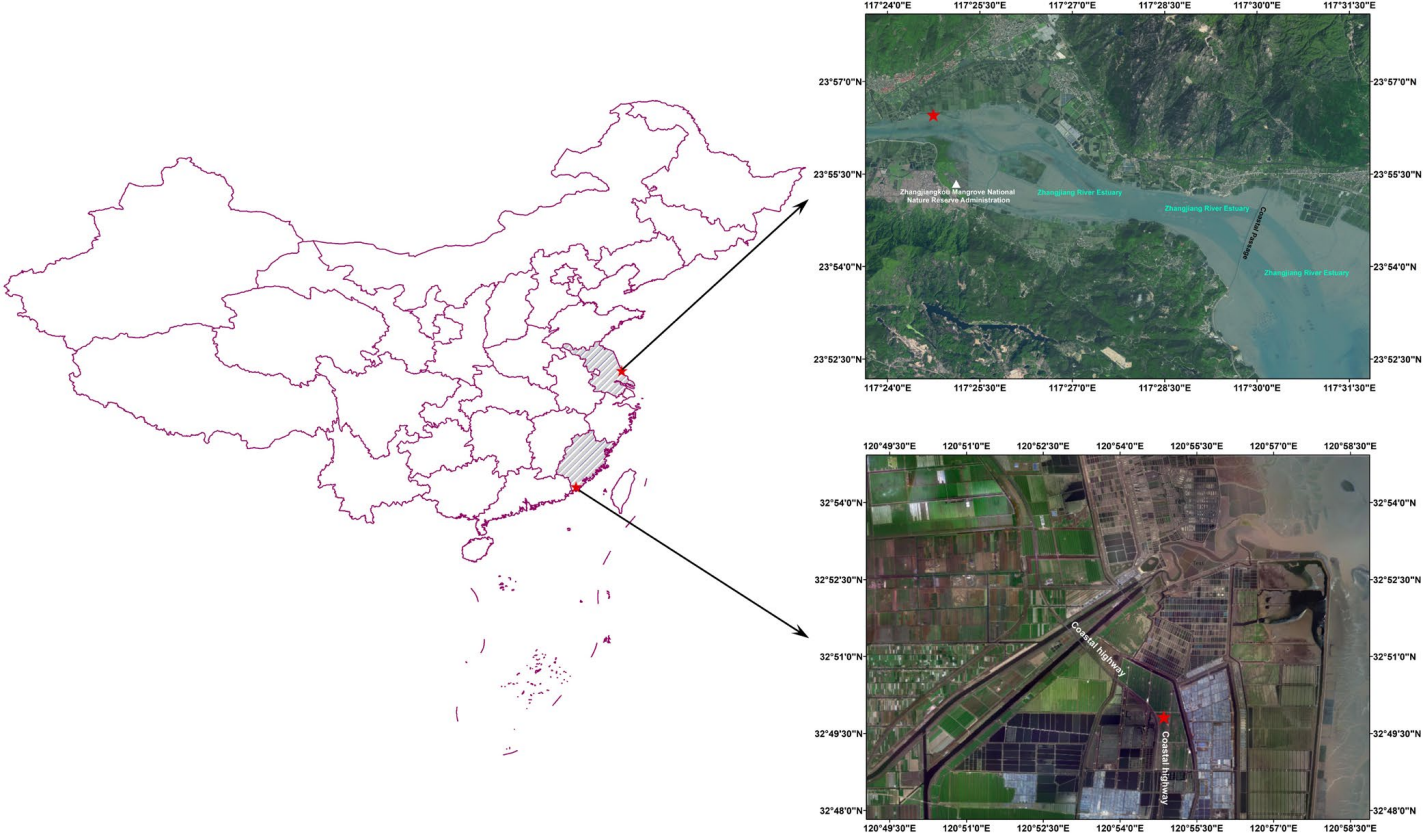
Study area. The red star was the location of sampling point.

The study area encompasses two distinct types of wetlands: the salt marsh wetland near the northwest of the Tiaozini wetland in Jiangsu (120°54′11.558″E, 32°49′34.171″N) and the Zhangjiangkou Mangrove Wetland Reserve in Fujian (117°24′07″–117°30′00″E, 23°53′45″–23°56′00″N), China (Figure 1). The Tiaozini wetland experiences a monsoon climate and is situated in the transition zone from the warm temperate zone to the north subtropical zone (Mo et al. 2023). It is the largest coastal wetland on the west coast of the Pacific Ocean and the edge of the Asian continent, falling within the Yellow Sea ecological area. With an annual average temperature of 14°C–15°C, summer precipitation comprises 40%–55% of the yearly rainfall. The north and south sides of the middle finger dam are populated by clusters or linear successions of the invasive plant species *S. alterniflora* , significantly reducing biodiversity and ecosystem functioning in the region and its surroundings. The study zone lies in a high beach arid area that is periodically submerged by high tides each month.

The Zhangjiang Estuary Mangrove Wetland Reserve experiences a subtropical maritime monsoon climate. The annual average temperature is 21.2°C, with an annual average precipitation of 1714.5 mm and an annual evaporation of 1718.4 mm. The predominant wetland type is a permanent mangrove wetland, featuring constructive species such as *Kandelia candel*, *Aegiceras corniculatum*, and *Avicennia marina* . The soil composition primarily consists of coastal beach silt and sandy silt (Mo et al. 2021).

In China, *S. alterniflora* is a dominant invasive species in salt marshes, and mangroves, as blue carbon ecosystems, are among the most important ecosystems on Earth. However, they are also increasingly threatened by the invasion of *S. alterniflora* , a problem that urgently needs to be addressed (Mo et al. 2021).

### 2.2 Sampling

The study was conducted in 2021, with sampling carried out across various growth stages of *S. alterniflora* to characterize the late‐stage development of the invaded ecosystems. A site within the study area featuring dense growth of *S. alterniflora* was selected for the experiment, and nine parallel quadrats of 40 m^2^ were established. A total of 72 soil samples were collected, with 36 samples from each site. Prior to sampling, soil volumetric water content (VWC) and conductivity (EC) of each quadrat were measured. Soil samples were collected at the green leaf stage, yellow leaf stage, withering stage, and fully withered stage of *S. alterniflora* . Samples collected at each stage were labeled JA, JB, JC, and JD in Jiangsu (or FA, FB, FC, and FD in Fujian), respectively.

Soil volumetric water content and conductivity were measured on‐site. Soil samples were collected from a depth of 0 to 20 cm at three random points in each parallel site and then mixed into one composite sample. In the laboratory, all visible roots were removed. Each soil sample was divided into three portions. The first part was stored at −20°C to determine ammonium nitrogen (NH_4_^+^‐N) and nitrate nitrogen (NO_3_^−^–N). The second part was air‐dried and sieved through a 1‐mm mesh to determine soil pH, total carbon (TC), total nitrogen (TN), total phosphorus (TP), soil organic carbon (SOC), and available phosphorus (AP). The final soil subsample was stored at −80°C for DNA extraction.

### 2.3 Soil Properties Analysis

In this study, a TOC analyzer (Vario TOC SELECT, Elementar, Germany) was used to directly determine soil TC. TOC content was measured using the potassium dichromate oxidation spectro‐optical method (Li et al. 2021). Soil TN was quantified using the Kjeldahl distillation method (Jia et al. 2018). SOC was determined using the potassium dichromate oxidation spectro‐optical method (Li et al. 2021). AP was measured using the NaHCO_3_ extraction–molybdenum antimony anti‐spectrophotometric method (Liu et al. 2020). Soil pH was measured using a pH/OPR/electrical conductivity/dissolved oxygen measuring instrument (SX751, SANXIN, Shanghai) at a ratio of 1:2.5 (*W/V*) (Liu et al. 2020). TP was assessed using the sodium hydroxide melting UV–visible spectrophotometry method (Zhang et al. 2018). NO_3_^−^‐N content was determined using the KCl extraction double‐wavelength UV colorimetric method, and NH_4_^+^‐N content was determined using the KCl extraction indophenol colorimetric method (Markus et al. 1985). All experiments were conducted in triplicate.

### 2.4 DNA Extraction, PCR Amplification, Sequencing, and Sequence Data Processing

The microbial community DNA was extracted using the NucleoSpin Soil Kit (Macherey–Nagel, Germany), following the manufacturer's instructions. DNA quantification was performed with a Qubit Fluorometer using a Qubit dsDNA BR Assay Kit (Invitrogen, USA), and quality assessment was conducted by running an aliquot on a 1% agarose gel.

The bacterial 16S rRNA gene in the variable regions V3–V4 was amplified using PCR primers: 341F (5′‐ACTCCTACGGGAGGCAGCAG‐3′) and 806R (5′‐GGACTACHVGGGTWTCTAAT‐3′). PCR amplification was carried out in a 50‐μL reaction containing 30 ng of template DNA, fusion PCR primer, and PCR master mix. The PCR cycling conditions were as follows: initial denaturation at 94°C for 3 min, followed by 30 cycles at 94°C for 30 s, 56°C for 45 s, 72°C for 45 s, and a final extension at 72°C for 10 min. Purification of PCR products was performed using Agencourt AMPure XP beads, followed by dissolution in an elution buffer. The libraries were qualified using the Agilent 2100 Bioanalyzer (Agilent, USA). Validated libraries were then subjected to sequencing on the Illumina MiSeq platform (BGI, Shenzhen, China), following standard Illumina pipelines and generating 2 × 300 bp paired‐end reads.

Off‐machine data reads were saved in FASTQ format and filtered to remove problematic sequences. The remaining high‐quality reads were combined into tags using the Fast Length Adjustment of Short Reads program (FLASH, v1.2.11) (Edgar 2013; Magoc and Salzberg 2011). The raw data were saved in FASTQ format and submitted to the NCBI BioProject database with the accession numbers: PRJNA1158092 (BioProject number). Tags were clustered into operational taxonomic units (OTUs) and compared with the library, and species were annotated by OTUs and ASVs based on DADA2 of QIIME2 (Mongad et al. 2021). Alpha and beta diversity were estimated using MOTHUR (Mongad et al. 2021) at the OTU level. Soil bacterial species abundance and diversity were assessed using diversity indices including OTU, Chao1, Shannon, Simpson (D), and ACE. KEGG functions were predicted using the PICRUSt software (Wilkinson et al. 2018). Sample species complexity analysis, intergroup species difference analysis, correlation analysis, and model prediction were based on the OTUs and annotation results.

### 2.5 Bacteriome Stability Index

In this study, we employed a novel method proposed and validated by Xun et al. (2021) to assess whole bacteriome stability. This method involves quantifying the stability index by calculating the average variation degree (AVD). Unlike some models, this approach is not limited by the number of samples within each group. Lower AVD values indicate greater microbial community stability (Xun et al. 2021). The variation degree of each OTU was calculated using Formula (1):

(1) $$\left|a_{i}\right|=\frac{x_{i}-{\bar{x}}_{i}}{\delta_{i}}$$

Where $a_{i}$ is the variation of OTUs, *xi* is the sparsity abundance of an OTU in a sample, ${\bar{x}}_{i}$ represents the mean sparsity abundance of OTUs in a sample group, and *δi* represents the standard deviation of rare abundance of OTUs in a sample group. The AVD value was calculated using Equation (2), where k is the number of samples in a sample group and n represents the number of OTUs in each sample group.

(2) $$\mathrm{AVD}=\frac{\sum_{i=1}^{n}\frac{|x_{i}-{\bar{x}}_{i}|}{\delta_{i}}}{k\times n}$$

### 2.6 Co‐Occurrence Networks and Stability Analysis of Bacterial Taxa

A valid bacterial taxa co‐occurrence correlation was established between each site and their D‐value of relative abundance based on the genus‐level data (including top 200 taxa and remaining taxa combined into a single group) if the Spearman's correlation coefficient (*r*) was greater than 0.6 with a *p*‐value < 0.01 (Liu et al. 2022a; Yuan et al. 2021). Subsequently, the topological characteristics of the entire network and each node were analyzed to evaluate the network's complexity. Topological characteristics, including average degree (avgK), which is a key property used to describe how well a node is connected to others, had a higher value in more complex networks; average clustering coefficient (avgCC), which was used to measure the extent of module structure in a network; average path distance (APD), which represented the average distance between every two nodes in a network, with a higher APD value indicating reduced coupling among nodes; modularity, which was calculated to measure how well a network could be separated into modules; and graph density (GD), which was closely related to the average degree. The degree (K) measured the connectivity of individual nodes, and nodes with high Hub and betweenness centrality indexes were considered vital in the network as they could reach other nodes quickly and efficiently. In the current study, nodes with the highest Hub (top5) and betweenness centrality (top5) were considered keystone taxa, with the node having the highest K value among them identified as the core taxa (Woodhouse et al. 2016). Among these, network size (i.e., total nodes and total links) usually refers to the total amount of diversity or functionality in a system and is the simplest descriptor of network complexity. Another widely used index of network complexity is avgK (Shen et al. 2023).

We further evaluated the stability of microbial networks by calculating robustness and maximum vulnerability (Herren and Mcmahon 2018; Yuan et al. 2021). To test the effects of taxa removal on the remaining taxa, the proportion of the remaining taxa in this network after random or targeted node removal (robustness) was calculated according to Herren and Mcmahon (2018). The vulnerability of each node measures its relative contribution to the global efficiency. The vulnerability of the network is represented by the maximum vulnerability of the nodes within it. The global efficiency of a graph was calculated as the average of the efficiencies over all pairs of nodes.

To verify the findings from the network analysis, we also calculated a different metric called cohesion (including positive, negative, and total cohesion), which is an abundance‐weighted, null model‐corrected metric based on pairwise correlations (Spearman correlation test) across taxa (Herren and Mcmahon 2018):

(3) $$\text{cohesion}=\sum_{i=1}^{m}{\text{abundance}}_{i}\times {\text{connectedness}}_{i}$$

where *m* is the total number of taxa in a community. In this study, the absolute values of the correlations are presented, and the sum of both absolute values represents total cohesion. Higher total cohesion indicates stronger community interaction and a more stable structure (Herren and Mcmahon 2018; Liu et al. 2022a).

### 2.7 Co‐Occurrence Network Analysis of Functional Gene Reads

The difference value (D‐value) of the relative abundance based on the reads of each functional gene category (KEGG functional gene annotated at KO level 2 and KO level 3) was calculated for co‐occurrence network analysis. All calculated gene categories were derived from broader categories (KO level 1 of KEGG functional gene annotation), and metabolic functions (KO level 2 and KO level 3) were further classified into broad or specialized metabolic functions based on relevant definitions (Xun et al. 2019). In this study, the functional traits of KO level 2, such as amino acid metabolism, carbohydrate metabolism, membrane transport, and metabolism of cofactors and vitamins, and the functional traits of KO level 3, such as base excision repair, pyruvate metabolism, and cysteine and methionine metabolism, were defined as “broad functions” The functional traits of nitrogen metabolism, sulfur metabolism, atrazine degradation, and xenobiotics biodegradation/metabolism at KO level 3 were considered “specialized functions” and were restricted to specific bacterial taxa (Funk 2020; Gianfreda and Rao 2008; Mus et al. 2016; Singh et al. 2014).

This study revealed extensive interconnections among functional categories, accompanied by substantial background noise. To mitigate the confounding effects of environmental factors and enhance the reliability of our findings, we established functional co‐occurrence correlation between the D‐value of gene categories (KEGG functional gene annotated at KO level 3) if the Spearman's correlation coefficient (*r*) was greater than 0.75 with a *p*‐value < 0.01 (Xun et al. 2019).

### 2.8 Statistical Analysis

A one‐way ANOVA and S‐N‐K test were conducted using SPSS 21.0. To evaluate the contribution of species richness and diversity to bacteriome stability, the relationship between AVD values and bacterial species richness and diversity was examined. R‐4.1.3 software was utilized to construct the data table required for co‐occurrence network analysis, as well as for calculating robustness vulnerability, and cohesion. The topological properties of the bacterial genus network and functional metabolic network were computed using Gephi software and visually represented. The relationship between AVD and keystone taxa was examined and redundancy analysis (RDA) was further performed to assess the significance of core communities to diversity. Regression and random forest models were implemented using R software to quantify the effects of key communities, core communities, and specialized functions on bacteriome stability and determine their relative contribution. All significance levels were set at *p* < 0.05. Additionally, the differences in all measured parameters between the two invaded wetland systems at each growth stage were used to characterize developmental variables during late‐stage succession.

## 3 Results

### 3.1 The Characteristics of Soil Physical and Chemical Properties

In all soil samples, higher levels of TN and NH_4_^+^‐N were observed in Fujian compared to Jiangsu, while NO_3_^−^‐N exhibited the opposite trend. Only TN showed a significant difference between the two regions (Table 1, *p* < 0.001). Regarding other soil properties, VWC, EC, TC, TOC, and AP were higher in Fujian than in Jiangsu (Table 1), whereas pH and TP showed the reverse pattern. Significant differences were noted between the two regions in terms of VWC (*p* < 0.001), EC (*p* < 0.001), pH (*p* < 0.001), TC (*p* = 0.001), TOC (*p* < 0.001), and AP (*p* < 0.001).

**TABLE 1:** Characteristics of D‐value in soil physicochemical properties between the two sites (Jiangsu‐Fujian).

Soil properties	Average value	Minimum value	Maximum value	Standard deviation	*p* (between two sites)
TN (g/kg)	−1.083	−1.539	−0.575	0.226	< 0.001
NH_4_^+^‐N (mg/kg)	−9.772	−51.800	27.100	21.111	0.053
NO_3_^−^‐N (mg/kg)	3.230	−0.128	14.087	3.442	0.051
VWC (%)	−51.436	−69.267	−38.6333	8.168	< 0.001
EC (μm/m)	−8355.290	−12,715	−5148.000	1652.526	< 0.001
pH	2.763	1.550	4.580	0.599	< 0.001
TC (g/kg)	−2.281	−12.100	10.400	4.825	0.001
TOC (%)	−1.314	−2.810	−0.030	0.681	< 0.001
TP (mg/kg)	7.028	−331.000	448.00	161.880	0.680
AP (mg/kg)	−20.776	−43.910	3.650	11.662	< 0.001

*Note:* Significant level: *p* < 0.05.

### 3.2 Differences in Soil Bacterial Diversity and Communities' Structure

A total of 3,550,890 16S rRNA sequences were obtained through MiSeq analysis of the 200–450 bp region from 72 soil samples collected across both sites. The average sequence numbers differed significantly between the two locations (Jiangsu‐Fujian) by 5679.667 (Table 2, *p* < 0.01), indicating a significantly higher quantity of soil bacteria in Jiangsu compared to Fujian, whereas the opposite trend was observed for OTUs. Specifically, the difference in OTU numbers between the two sites was −3055.42. Moreover, the Chao1, Shannon, and ACE indices for soil from Fujian were all significantly higher than those from Jiangsu (Table 2, *p* < 0.01), whereas the Simpson (D) index was higher in soil from Jiangsu, suggesting that the bacterial species diversity of soil from Fujian was significantly greater than that from Jiangsu.

**TABLE 2:** D‐value of abundance and diversity between two sites (Jiangsu‐Fujian).

	Tag	OTU	Chao	ACE	Shannon	Simpson (D)
Average value	5679.667**	−3055.42**	−4826.68**	−5849.340**	−1.300**	0.0200**
Standard deviation	6494.014	743.798	1352.062	1877.606	0.574	0.021
Minimum value	−6509.000	−3898.33	−6773.330	−8587.550	−2.422	0.0006
Maximum value	18599.670	−1386.670	−1833.140	−1963.390	−0.362	0.072

** *p* < 0.01.

**FIGURE 2:**
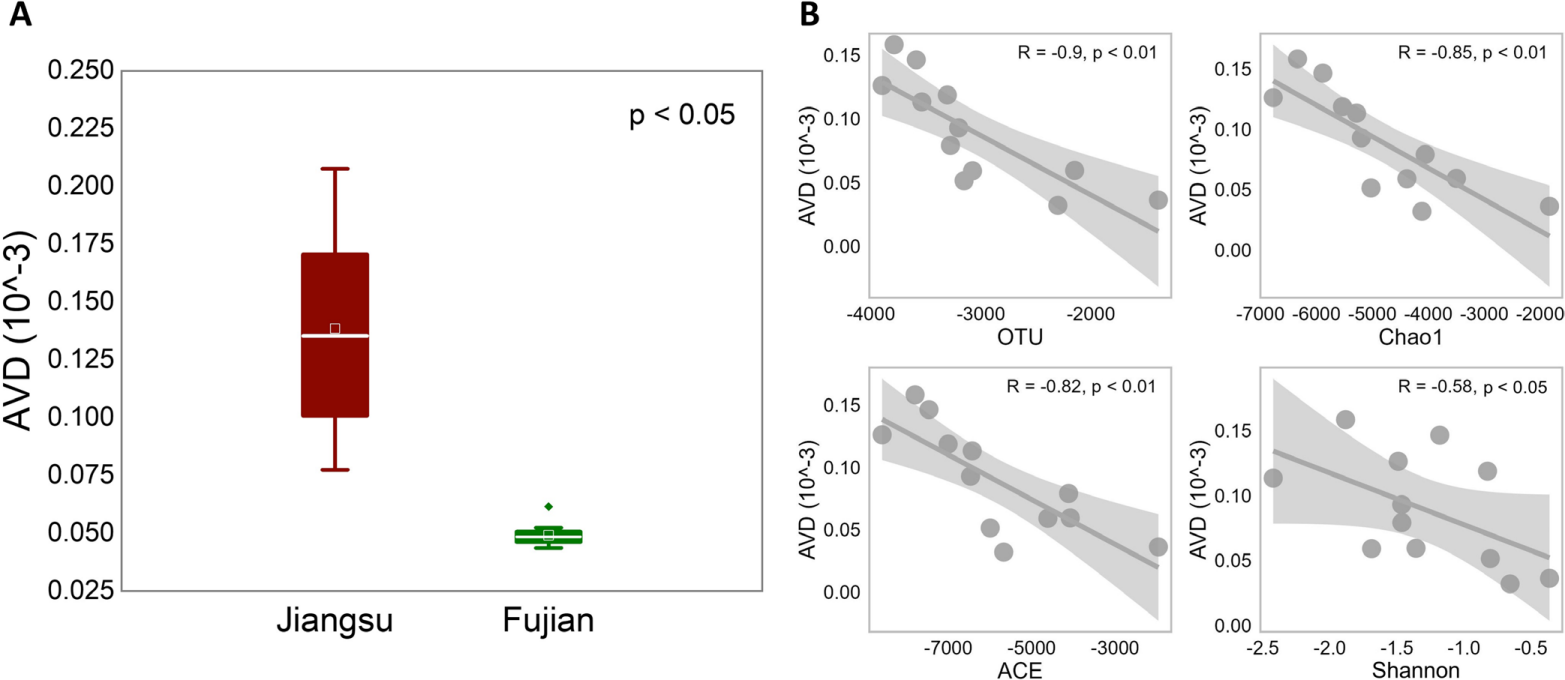
AVD characteristics of two sites and their relationship with soil bacterial diversity.

The site‐specific AVD calculations revealed significantly lower values in Fujian soil (Figure 2A, *p* < 0.05). Furthermore, a significant negative linear correlation was observed between AVD values and species richness (Figure 2B, OTU, *p* < 0.001; Chao1, Figure 2C, *p* < 0.001), as well as diversity (Shannon, Figure 2D, *p* < 0.05; ACE, Figure 2E, *p* < 0.01). Additionally, AVD and diversity at the initial stages of the two sites were significantly associated with wetland environmental backgrounds (soil properties and vegetation, *p* < 0.05), except for NH_4_^+^‐N and TP (*p* > 0.05). However, during late‐stage development, only NO_3_^−^‐N (negative correlation) and EC (positive correlation) were significantly associated with diversity, while other parameters showed no correlation with AVD or diversity (Figure S1).

### 3.3 Bacterial Community Structure and Keystone Taxa

**FIGURE 3:**
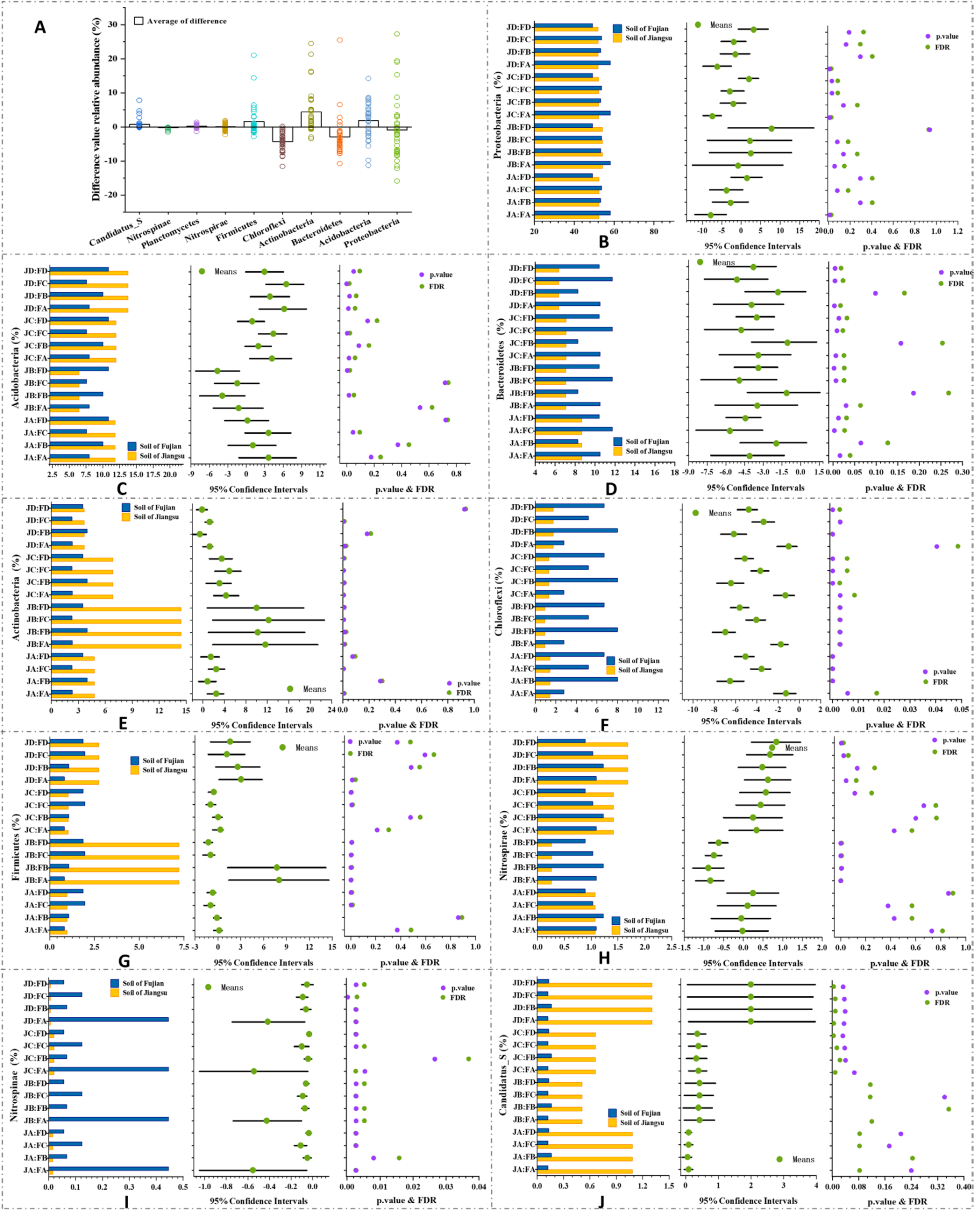
D‐value in relative abundance of dominant phyla and phyla with significant difference between the two sites (Jiangsu and Fujian). In Figure F–J, “FA”, “FB”, “FC”, “FD” represent soil samples of Fujian, “JA”, “JB”, “JC”, and “JD” represent soil samples of Jiangsu; the left panel shows the average relative abundance of phyla in each group, the middle panel shows the log2 value of the ratio of the average relative abundance of the same phylum between the two sites, and the right panel shows the p‐value and FDR values based on the Wilcoxon test, with differences considered significant when both p‐value and FDR are less than 0.05. A: Colored circles represent the difference in relative abundance of each phylum between soil samples corresponding to the two sites. B: Proteobacteria. C: Acidobacteria. D: Bacteroidetes. E: Actinobacteria. F: Chloroflexi. G: Firmicutes. H: Nitrospirae. I: Nitrospinae. J: Candidatus_S (Candidatus_Saccharibacteria).

The top 10 phyla (with an average relative abundance > 0.5%) based on the total 16S bacterial data from all samples were identified (Figure 3) as Proteobacteria (53.137%), Acidobacteria (10.984%), Bacteroidetes (7.287%), Actinobacteria (3.028%), Chloroflexi (1.339%), Firmicutes (1.439%), Nitrospirae (1.062%), Planctomycetes (0.399%), Nitrospinae (0.170%), and Candidatus_Saccharibacteria (0.133%). Proteobacteria was identified as the predominant phylum (*p* < 0.01). The taxonomic group structures of soil bacteria between the two sites differed significantly, as indicated by the D‐value of each dominant phylum; however, only Planctomycetes showed no significant difference (Figure 3, *p* > 0.05). Analysis of these dominant phyla's influence on bacteriome stability and diversity during system development revealed that Proteobacteria and Nitrospinae showed significant positive correlations with AVD, and Proteobacteria was significantly negatively correlated with both Chao1 and ACE indices, indicating their expansion increases AVD and reduces stability. Moreover, both rare taxa and Nitrospirae exhibited significant positive correlations with diversity indices (Table S1).

**FIGURE 4:**
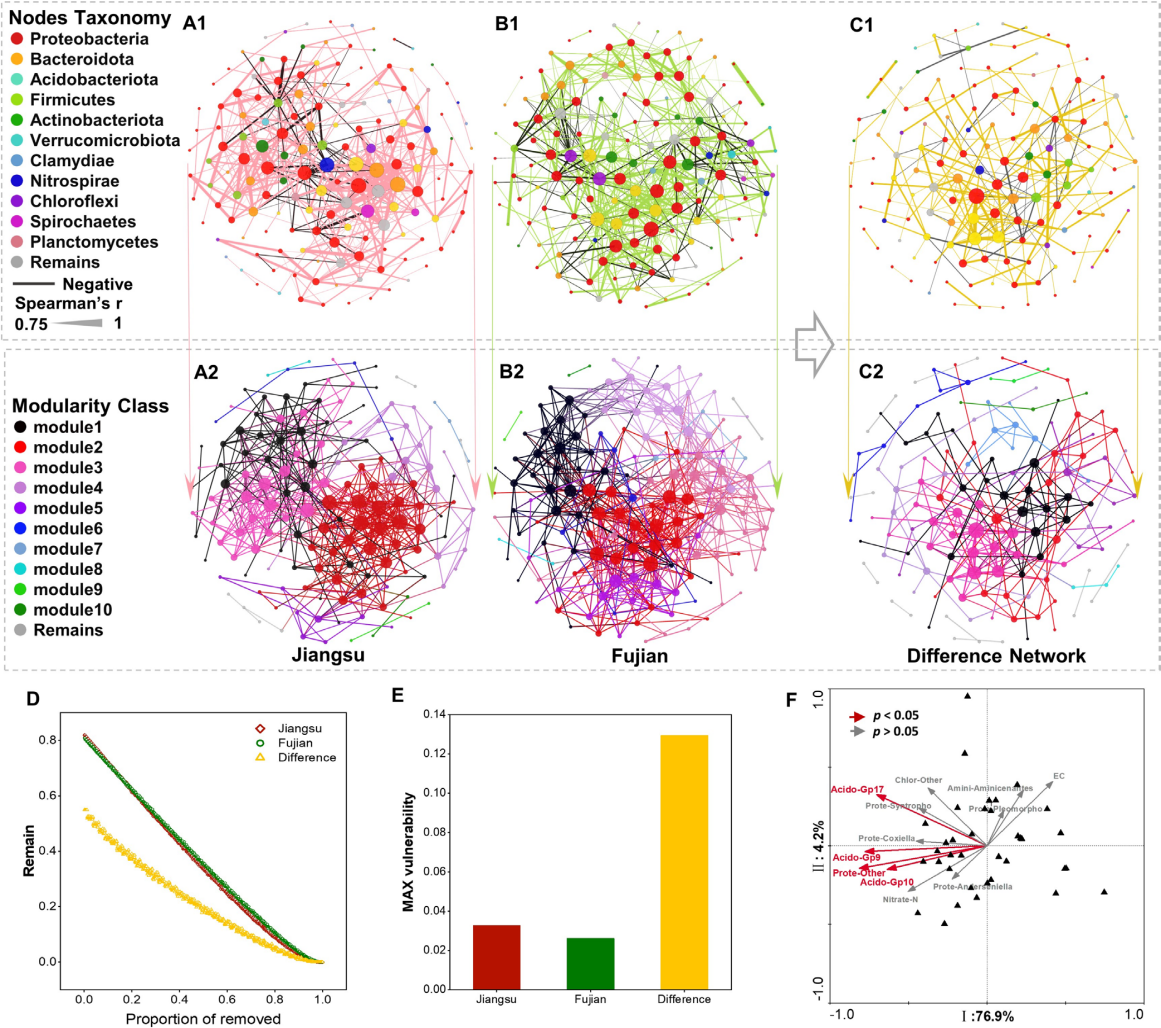
Co‐occurrence networks and ability of bacterial genera at Jiangsu and Fujian and different (D‐value) of the two sites; and the impact of keystone taxa on diversity. In A, B, C, Node size represents the degree of node connectivity; each dashed box represents a module. In A1, B1, C1, different colors of nodes represent different phyla; in A2, B2, C2, different colors of nodes and edges represent different modules. In F, IR: Importance ranking. Prote‐Other: Proteobacteria‐Other. Acido‐*Gp17*: Acidobacteria‐*Gp17*. Acido‐*Gp9*: Acidobacteria‐*Gp9*. Acido‐Gp10: Acidobacteria‐Gp10. Firmi‐*Coxiella*: Firmicutes‐*Coxiella*. Prote‐*Anderseniella*: Proteobacteria‐*Anderseniella*. Prote‐*Syntrophobacter*: Proteobacteria‐*Syntrophobacter*. Amini‐*Aminicenantes*: Aminicenantes‐*Aminicenantes*. Proteo‐*Pleomorpho*: Proteobacteria‐*Pleomorphobacterium*. Acido‐Other: Acidobacteria‐Other. D: Robustness. E: Maximum vulnerability.

The genus‐level co‐occurrence networks of the top 200 taxa exhibited differences in soil bacterial co‐occurrence networks between Jiangsu and Fujian, as well as between the two sites (Figure 4). Fujian exhibited higher network complexity and connectivity (Figure 4). The Fujian network demonstrated greater structural stability, characterized by the highest number of edges (626) and avgK (8.29). Despite having a higher proportion of negative correlations (21.41%), the network showed shorter APD and higher GD, indicating a more compact structure (Figure 4A–C and Table S2). Both wetlands exhibited significant modularity in their networks (Figure 4A2,B2,C2 and Table S2). The bacterial network in Jiangsu showed higher modularity but lower avgCC compared to Fujian. Significant differences in modular composition between the two networks indicate distinct microbial community structures associated with each invaded wetland. Network robustness revealed comparable resistance and cohesion in dominant genera structures at both sites. However, the Fujian network demonstrated lower maximum vulnerability and superior robustness with increasing node removal compared to Jiangsu (Figure 4D–F). These findings further support the higher stability of soil bacterial communities in Fujian's mangrove invasion system relative to Jiangsu's salt marsh.

The differential network reflects the differences in bacterial community structure during the later stages of development between the two systems, comprising 143 nodes and 249 edges, with an avgK of 3.48, avgCC of 0.35, APD of 5.04, modularity of 0.71, GD of 0.025, and a negative correlation ratio of 16.87%. Eighteen modules were identified based on the topological features of individual nodes, and the network's core groups were determined (Table 3). The ubiquity of genera in Proteobacteria across all modules was demonstrated in the network, with the highest complexity found in Module 1 (17.48%), and most taxa in the top four modules (each ratio > 50%, Table S3) belonging to Proteobacteria. Additionally, Bacteroidetes was the other main bacterium in Module 2 (16.78%), while Acidobacteria were the other main bacteria in Module 3 (15.38%) and Module 4 (10.49%).

**TABLE 3:** The keystone taxa of differential network and their correlation with AVD.

Node	Taxonomy	Degree	Betweenness centrality	Hub	Spearman's *r*
					AVD
Other	Proteobacteria	16	673.852	0.397	N
*Gp17*	Acidobacteria	13	785.120	0.337	N
*Gp10*	Acidobacteria	12	545.223	0.303	N
*Gp9*	Acidobacteria	11	336.880	0.284	N
*Coxiella*	Firmicutes	9	89.057	0.188	N
*Anderseniella*	Proteobacteria	6	1495.650	0.038	N
*Syntrophobacter*	Proteobacteria	6	1430.059	0.006	N
*Aminicenantes*	Aminicenantes	5	1002.371	0.001	−0.776**
*Pleomorphobacterium*	Proteobacteria	6	930.319	0.083	N
Other	Acidobacteria	6	785.120	0.012	N

** *p* < 0.01.

The differential network identified 10 nodes with the top 5 Hub and betweenness centrality as keystone taxa. Among these, the core genus was defined as the node with the highest degree (K = 16), which consisted of rare genera within Proteobacteria (Other, K = 16) (Table 3). Notably, *Aminicenantes* showed a significant negative correlation with AVD, suggesting its direct role in enhancing bacterial community stability. Redundancy analysis (RDA) was performed to rank the relative importance of keystone taxa and significant abiotic factors (EC and NO_3_^−^‐N) influencing bacterial diversity. The results revealed that the explanatory amounts of keystone taxa on Axis I and Axis II were 76.9% and 14.2%, respectively, which accounted for a total of 91.1% of the variation. The significant contributors (*p* < 0.05) to bacterial diversity were ranked in order of importance as Other (Proteobacteria), Gp17, Gp9, and Gp10 (Figure 4F and Table S4). Notably, all keystone taxa belonged to the predominant phyla identified within large modules and exhibited significantly lower influence on diversity compared to biotic factors.

**FIGURE 5:**
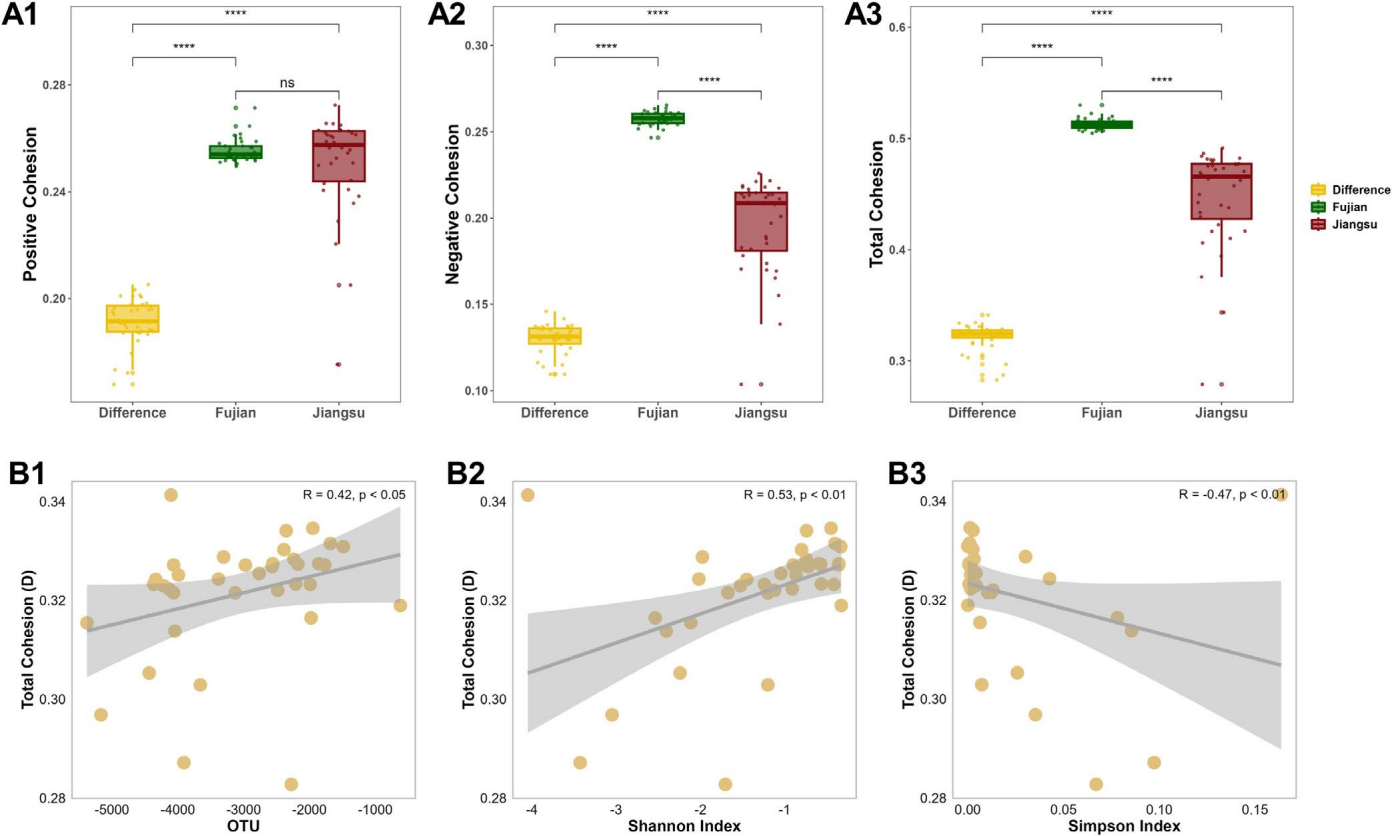
Cohesion of bacterial genus in Jiangsu and Fujian and difference (D‐value) between the two sites, the effect of diversity on the total cohesion (D‐value based). A1: Positive cohesion; A2: Negative cohesion; A3: Total cohesion. B1: Effect of total cohesion on OTU; B2: Effect of total cohesion on Shannon index; B3: Effect of total cohesion on Simpson (D) index.

The wetland in Fujian exhibited slightly higher positive cohesion compared to Jiangsu (Figure 5A1), while both negative and total cohesion were significantly higher in Fujian (Figure 5A2,A3), indicating a more stable genus structure in Fujian, consistent with the AVD results. Further analysis revealed that soil physicochemical properties did not drive the differences in total cohesion of bacterial taxonomic groups between the two sites (Figure 5B1–B3). However, total cohesion showed significant positive correlations with OTU and Shannon, and a significant negative correlation with Simpson, suggesting that increased diversity contributes to the stability of the community structure.

### 3.4 The Impact of Different Levels of Metabolic Function on AVD

**FIGURE 6:**
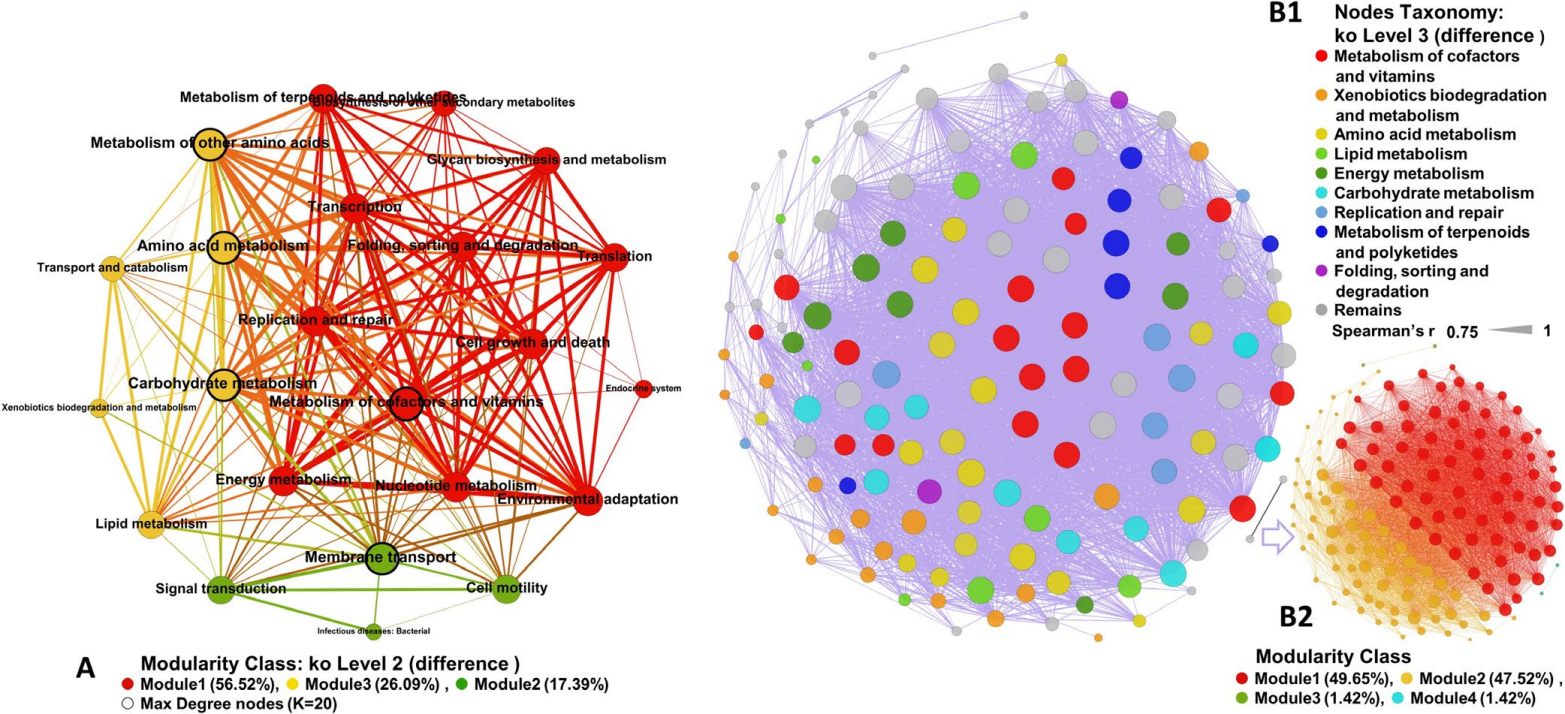
The co‐occurrence networks of the metabolic functional reads in the two sites (based on D‐value of Jiangsu and Fujian). In A, modules are divided by color, red nodes: Module1, green nodes: Module2, yellow nodes: Module 3; blue nodes: Module4. In B1, different color of nodes represents different metabolic function of KEGG KO level 2. In B2, a color represents a module.

After filtering for a correlation coefficient of r > 0.75 and *p* < 0.01, a bacterial functional co‐occurrence network model was constructed based on KEGG KO level 2 and level 3 metabolic function reads, which revealed differences between the two sites (Figure 6). In this study, as the functional network contained numerous key nodes, only the core functions within the key functions were analyzed for their contribution to bacteriome stability. The metabolic functional network at level 2 consisted of 23 nodes and 187 edges (Figure 6A and Table S5), with five core functions having the highest connectivity (K = 20), including amino acid and other amino acid (total amino acid) metabolism, carbohydrate metabolism, membrane transport, and metabolism of cofactors and vitamins. The network was divided into three modules, with module 1 containing the most core metabolic functions, which played a critical role in linking the other modules.

The network at level 3 displayed high complexity, with 141 nodes and 4373 edges (Figure 6B1,B2 and Table S5). The core metabolic functions with the highest node connectivity (K = 96) were base excision repair, cysteine and methionine metabolism, and pyruvate metabolism. The network was divided into five modules, with modules 1 (49.65%) and 2 (47.52%) accounting for a total of 91.17% of metabolic functions (Figure 6, Table S6). Most nodes with high connectivity (K > 50) were found in module 1, which included the sulfur relay system and methane metabolism. Module 2 contained all core metabolic functions, including specialized functions such as nitrogen metabolism, sulfur metabolism, metabolism of xenobiotics by cytochrome P450, polycyclic aromatic hydrocarbon degradation, and atrazine degradation. Only 47.76% of nodes in this module had a K > 50%.

**FIGURE 7:**
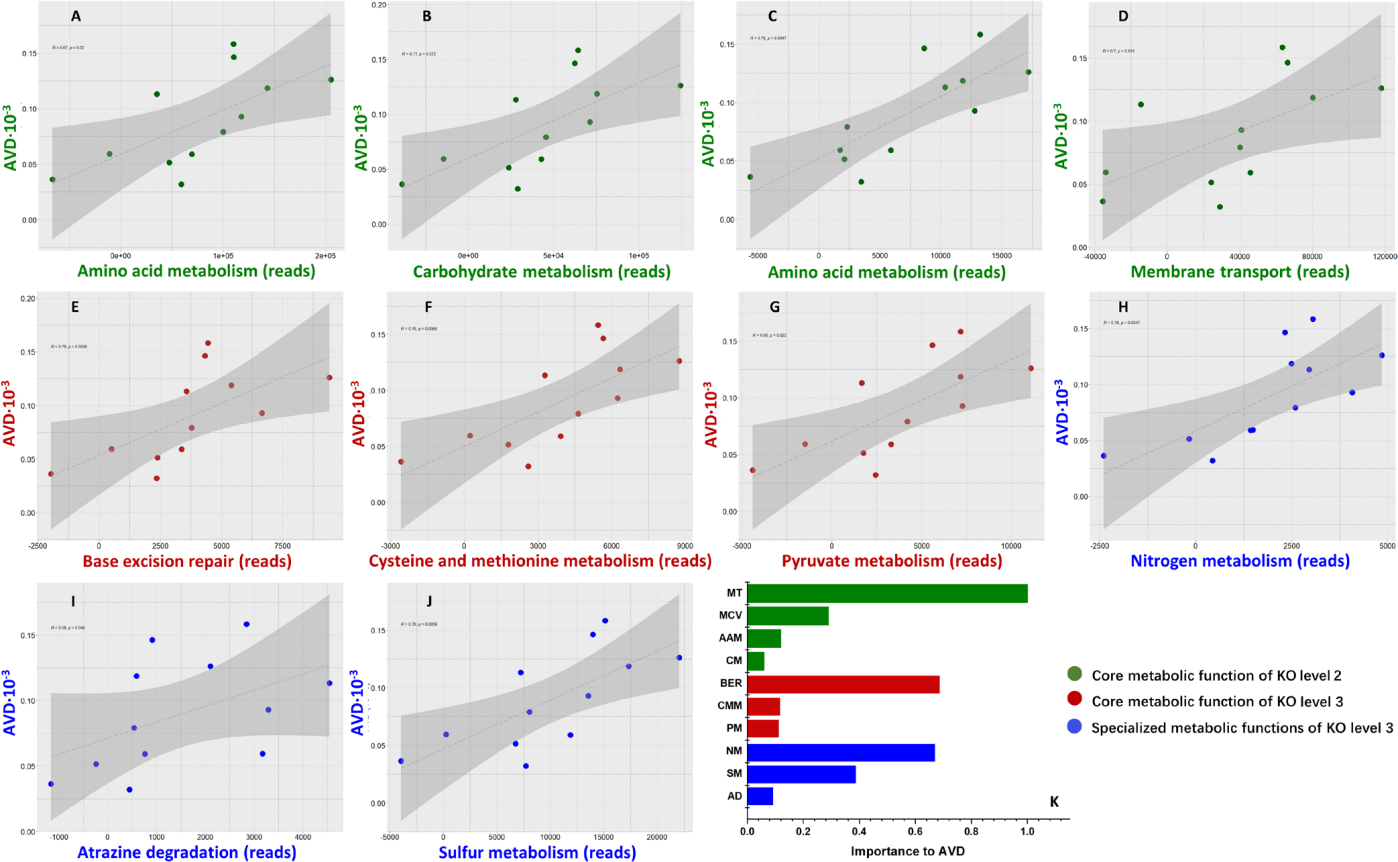
The impacts and contribution of two levels of core and specialized metabolic functions to AVD changes. AAM (A): Amino acid metabolism. CM (B): Carbohydrate metabolism. MT (C): Membrane transport. MCV (D): Metabolism of cofactors and vitamins. BER (E): Base excision repair. CMM (F): Cysteine and methionine metabolism. PM (G): Pyruvate metabolism. NM (H): Nitrogen metabolism. AD (I): Atrazine degradation. SM (J): Sulfur metabolism.

The influence of microbial stability on microbial function was further explored (Figure 7), revealing significant positive correlations between AVD and all core functions of level 2 (total amino acid metabolism, carbohydrate metabolism, membrane transport, and metabolism of cofactors and vitamins) (Figure 7A–D), as well as the core functions of level 3. Relationships between six specialized functions and bacterial stability were further analyzed, uncovering that nitrogen metabolism (Figure 7H, *p* = 0.0047), sulfur metabolism (Figure 7J, *p* = 0.0059), and atrazine degradation (Figure 7I, *p* = 0.046) significantly positively influenced AVD. The results suggest that enhancing these functions decreases the stability of the bacterial structure. The importance values of these functions on AVD were predicted by random forest analysis, revealing that membrane transport (Figure 7K) was the largest contributor among all the above functions to the stability of the microbial community (28.33%). In the level 3 network, the impact contributions of nitrogen metabolism (NM, specialized function) (18.93%) and base excision repair (BER, broad function) (19.48%) to AVD were similar and second to membrane transport. It was also evident that specialized functions had a greater impact on stability than broad functions.

## 4 Discussion

### 4.1 Influence of Abiotic Factors on Soil Bacterial Diversity and Bacteriome Stability

Abiotic factors (such as hydrology and soil properties) determine the foundational structure of soil bacterial functional groups (Loreau 2001). In our study, the environmental baseline shaped the soil bacterial community structure in the invaded wetland systems, establishing the foundation for stability. However, in the later‐stage development of these two systems, the differences between their progression were primarily driven by bacterial (biotic) factors. First, the hydrological conditions differed significantly: the mangrove wetland invaded by *S. alterniflora* (Fujian study area) experiences daily wet‐dry alternation, whereas the Tiaozini salt marsh invaded by *S. alterniflora* (Jiangsu study area) is submerged only during spring tides. This results in a higher proportion of hydrophilic bacterial taxa in mangrove soil compared to salt marsh, contributing to differences in bacterial community structure. Crucially, mangrove sediments exhibited significantly higher organic matter and nutrient content (Table 1) and consisted of extremely fine particles, giving them excellent water retention and nutrient‐holding capacity. This supports the high stability and diversity of the bacterial community (Albaladejo et al. 2013). Moreover, the invasion of *S. alterniflora* has further complicated the soil environment (Mo et al. 2021). Conversely, in the Jiangsu study area, where few native plants remain in the vicinity, the soil environment is relatively simplified (Chen et al. 2004).

Bacterial diversity tends to increase with greater environmental complexity and nutrient richness (Erkus et al. 2013; Shu and Huang 2022). Consequently, soil bacterial characteristics, including diversity and structure, vary significantly between the two sites with contrasting basal conditions (Table 2). However, in this study, soil physicochemical properties were not the driving force behind the differences in stability trends during the later‐stage development of the two invaded ecosystems. The comparable robustness, maximum vulnerability, and positive cohesion of genus‐level networks under distinct environmental conditions further support this conclusion (Figures 4 and 5; Table S2). In the later‐stage development of the invaded systems, we found a significant negative correlation between soil NO_3_^−^‐N and bacterial diversity (Figure S1). This relationship is attributed to the historical application of nitrogen‐containing herbicides in both regions, leading to elevated nitrate levels, which subsequently inhibited bacterial proliferation and metabolic activity (Mo et al. 2021). Notably, bacterial stability and diversity were more strongly regulated by interspecies interactions than by abiotic factors. This is because the development of wetland systems successfully colonized by *S. alterniflora* tends to stabilize. For instance, gradual processes such as carbon sequestration establish a dynamic equilibrium, creating environmental conditions that remain within the self‐regulatory capacity of bacterial communities. This allows bacteria to continuously participate in material cycling and energy flow within the habitat. During this process, bacterial diversity is maintained due to the absence of abrupt environmental stressors, thereby further ensuring the stability of the bacterial structure. This resilience may be attributed to the inherent physiological and genetic characteristics of bacteria, enabling them to express adaptive genes and maintain internal environmental stability in response to varying conditions (Tardy et al. 2014).

### 4.2 Influences of Soil Bacterial Diversity and Core Bacteria on Stability

The relationship between biodiversity and ecosystem stability in microbial ecosystems has long been debated in theoretical ecology. However, due to the high complexity, diversity, and variability of soil microbial communities, there have been limited experiments exploring this ecological theory in soil microbial ecosystems, and research utilizing interaction models is progressing slowly (Coyte et al. 2015). They concluded that diversity serves as a robust guarantee for microbial community stability (Loreau 2001; Mccann 2000; Tardy et al. 2014). This partly explains the observed high stability in Fujian soil (low AVD, Figure 2), and the relationship between diversity and AVD supports the notion that diversity drives bacteriome stability. In our study, the differences in the changes in bacterial community stability and diversity during the later‐stage development of the two *S. alterniflora* invasion systems were significant (Figure 2 and Table 2), with diversity ensuring the maintenance of a functional microbial community under changing environmental conditions. The insurance hypothesis, proposed earlier, suggests that higher diversity in a community may contain more complementary functional groups adapted to specific stressors, which typically increases the functional diversity and complementary functions of the community, thereby reducing the risk of system function loss, ensuring stronger restoration ability, and enhancing the resilience of the community after experiencing stress (Yachi and Loreau 1999). Moreover, higher bacterial diversity implies a greater number of interspecies interactions and stronger niche differentiation, which reduces direct competition among species and improves resource utilization efficiency, thereby influencing the robustness of biogeochemical cycling processes within the system.

Similarly, the bacterial genus network in the Fujian study area exhibits higher complexity than in the Jiangsu study area, with more edges and avgK, as well as a higher avgCC. This, to some extent, promotes bacteriome stability (Shen et al. 2023), and the total cohesion characteristics of the bacterial communities further support the finding that the bacterial community structure in Fujian soils exhibited the highest stability. Moreover, the higher proportion of negative correlations and negative cohesion indicates more competitive and antagonistic interactions in Fujian, which is key to high diversity (Tardy et al. 2014). In addition to the influence of native mangrove plants, this is also attributed to the overwhelmingly dominant role of positive connectivity in all three networks. Under such conditions, interspecies interactions (e.g., competition, predation, and mutualism) are crucial in maintaining ecological bacteriome stability, thereby enhancing resistance to disturbance (Coyte et al. 2015; Stone 2020).

Among the top ten phyla, Proteobacteria overwhelmingly dominated the community, and significant changes were found between the two *S. alterniflora‐*invaded systems during their later developmental stages for all taxa except Planctomycetes, suggesting that these phyla may be keystone taxa driving soil bacteriome stability throughout the growth process of *S. alterniflora* . The impact of these taxa (the most abundant OTUs) on maintaining microbial community function in the lake was evaluated by Garcia‐Garcia et al. (Garcia‐Garcia et al. 2019). They showed that the communities were significantly driven by bacteriome stability, ensuring the maintenance of stable community function. In the co‐occurrence network of the top 200 genera in the bacterial variables between the two sites (Figure 4), a large proportion of the taxa belong to these phyla, and taxa belonging to Proteobacteria are dominant in each module of the network. Simultaneously, 90% of the keystone taxa belong to the dominant phyla Proteobacteria, Acidobacteria, and Firmicutes, which align with the findings of Garcia‐Garcia. Due to their dominance, these taxa perform most of the ecological functions during the growth of *S. alterniflora* . Through mechanisms such as functional complementarity, niche differentiation, collective cooperation, and resource sharing, they maintain functional redundancy within the community, thereby ensuring its fundamental stability.

Liu et al. (2022b) found that the number of keystone taxa showed a similar trend with network complexity and stability, and their disappearance may lead to the collapse of modules and the network (Coux et al. 2016; Xue et al. 2018), indicating that networks containing more potential keystone taxa are more stable. In this study, multiple keystone taxa within Proteobacteria and Acidobacteria were found to significantly influence bacterial diversity and offset the impact of abiotic factors (Figure 4 and Table 3). This confirms that keystone taxa directly or indirectly drove soil bacteriome stability during the growth of *S. alterniflora* . The unique and significant roles played by specific microorganisms in community structure and function have been demonstrated in numerous studies, in addition to the dominant taxa in microbial communities (Liu et al. 2022a; Ma et al. 2020b). We identified *Aminicenantes*, a keystone taxon belonging to the rare phylum Aminicenantes, as a direct contributor to bacteriome stability despite its low abundance (Table 3). Through niche differentiation, they utilize resources inaccessible to dominant species, filling specific ecological functional gaps (Herren and Mcmahon 2018; Xun et al. 2021).

### 4.3 Influences of Functions on Bacteriome Stability

The core and specialized functions represent critical mechanisms in bacterial adaptation to environmental changes, serving as the main driving forces of bacteriome stability. During different growth stages of *S. alterniflora* , the developmental differences of the Level 3 functional network between the two sites exhibited high complexity, as indicated by the links and avgK in the Level 3 network (Figure 6 and Table S5). This arises from the functional redundancy of soil bacterial communities, implying that a moderate reduction in the abundance of any taxon may have minimal impacts on the overall function of the soil microbial community, as other taxa can perform similar functions (Huang et al. 2021; Kembel et al. 2010).

However, in this study, significant functional abundances negatively affecting stability were found to exhibit a positive feedback mechanism with AVD, suggesting that increased metabolic activity of core and specialized functions during the late‐stage development of invaded wetland systems reduced community stability. The core functions of the Level 2 network were common “broad functions” that typically ensure the survival and genetic functions of bacteria. For instance, membrane transport, which has the greatest impact on stability (Figure 7K), facilitates the transportation of substances within bacterial cells and maintains normal nutrient transport (John 1993). Additionally, amino acid metabolism, carbohydrate metabolism, and metabolism of cofactors and vitamins serve as key metabolic pathways coordinated to maintain bacterial cellular homeostasis and provide necessary components for proper cellular function while ensuring the energy status of the cell (Ghouili et al. 2023). Specialized functions like sulfur metabolism, nitrogen metabolism, and atrazine degradation are present in a minority of taxa (such as *Nitrospira*), yet they are crucial for maintaining the nitrogen and sulfur cycles and degrading harmful substances in ecosystems (Gao et al. 2019; Ladd et al. 1993; Singh and Schwan 2011; Siripattanakul et al. 2009). The study by Xun et al. (2021) showed that the specialized metabolic functions of keystone taxa maintained the stability of soil microbial communities, as assessed by constructing a co‐occurrence network (Xun et al. 2021). These findings collectively highlight the essential role of such functions in preventing ecosystem functional collapse and ensuring its sustained operation. However, this contradicts the hypothesis of this study.

However, all the above findings contradict the hypothesis of this study, as their increase instead led to a decrease in stability. It suggests that these key functional traits may be critical factors in reducing the stability of the soil bacterial community during the growth of *S. alterniflora* . This phenomenon can be attributed to the effect of cooperative destabilization, where coupling between species and positive feedback loops are induced by cooperative relationships. In this study, the negative rates of the functional network at both levels were close to zero, indicating a predominantly cooperative network structure (Figure 7) that significantly reduces bacteriome stability (Figure 7). In an almost entirely cooperative relationship, species are easily subject to dependency (Paine 1966). The reduction of one species can bring about a decrease in others, causing a decline in system stability (Coyte et al. 2015; Paine 1966). In severe cases, cascading collapse may occur, ultimately leading to total extinction (Dunne and Williams 2009). Collaboration means creating dependencies. The excessive cooperation‐dominated relationship carries the risk of functional redundancy loss and functional excess, where a highly efficient metabolic rate comes at the cost of reducing ecological stability and has the potential for mutual collapse (Coyte et al. 2015).

Such functional processes may also destabilize the overall community by triggering the accumulation of metabolic byproducts or competitive metabolic pathways. For example, amino acid metabolites (such as vitamins and amino acids) can serve as public resources, facilitating the cooperation of certain complementary bacterial groups. However, the stability of the entire system may decline if the dominant bacterial groups are disturbed by environmental changes. In future management strategies for *S. alterniflora* , it will be worth considering the promotion of bacterial communities associated with related functions to reduce the stability of the soil bacterial community in *S. alterniflora* ‐invaded wetlands, thereby inhibiting its growth.

## 5 Conclusion

Our findings indicate that higher bacterial community diversity in the later‐stage development of *S. alterniflora* invasion systems correlates with greater bacteriome stability, with biotic factors exerting a stronger influence on stability than abiotic factors. Furthermore, both core functions (broad functions) and specialized functions (such as “nitrogen metabolism” and “sulfur metabolism”) are significant in determining the stability of the bacterial community, and an increase in their metabolic activity can reduce bacteriome stability. This study offers additional insights into the relationship between soil microbial community biodiversity and ecosystem stability, highlighting the critical roles that keystone taxa and functional traits may play in maintaining soil microbiome stability. The findings are essential for understanding how keystone taxa, functional traits, and nitrate nitrogen regulation directly or indirectly influence the diversity and stability of soil bacterial communities, disrupting the stability of *S. alterniflora* ‐invaded systems, thereby offering a scientific basis for the management and restoration of invaded ecosystems.

## Author Contributions

**Xue Mo:** conceptualization (equal), data curation (lead), formal analysis (lead), investigation (equal), methodology (lead), validation (lead), visualization (lead), writing – original draft (lead), writing – review and editing (lead). **Zhenming Zhang:** conceptualization (equal), supervision (lead). **Yinglong Chen:** writing – review and editing (lead). **Shijun Zhou:** investigation (equal). **Yi Li:** investigation (equal). **Siqi Zhao:** investigation (equal). **Xuanming Chen:** investigation (equal). **Bo Wu:** investigation (equal), resources (equal). **Shiqiang Zhao:** investigation (equal). **Mingxiang Zhang:** conceptualization (equal), funding acquisition (equal), project administration (equal), resources (equal), supervision (equal).

## Conflicts of Interest

The authors declare no conflicts of interest.

## Supporting information


**Figure S1.** The effect of soil physicochemical properties on bacterial diversity and average variation degree. * indicate significant differences between soils in two sites, with *p* < 0.05.
**Table S1.** The relation between bacterial dominant phylum and diversity.
**Table S2.** The topological properties of bacterial genus co‐occurrence networks.
**Table S3.** Module scores and major taxa abundance in the hold three bacterial genus networks.
**Table S4.** Importance ranking of factors that significantly affect genus structure.
**Table S5.** The topological properties of the metabolic functional co‐occurrence networks on two KEGG ko levels.
**Table S6.** Module scores and major taxa abundance in the functional differential networks.

Data S1.

## Data Availability

All data relevant to the study are made available in Supporting Informations. Additionally, the 16S rRNA gene amplicon sequencing data in the current study have been deposited in the NCBI BioProject database under the accession number PRJNA1158092 (https://www.ncbi.nlm.nih.gov/sra/PRJNA1158092).
