# Supplementary material for: Spartina alterniflora Ecosystem Stability: Insights Into the Interplay Between Soil Bacteria and Their Functional Traits
Source: Ecol Evol. 2025 Apr 3;15(4):e71096. doi: 10.1002/ece3.71096 (PMC11968255; doi:10.1002/ece3.71096)
Supplement: Supplementary file 1 — Figure S1. The effect of soil physicochemical properties on bacterial diversity and average variation degree. * indicate significant differences between soils in two sites, with p < 0.05. Table S1. The relation between bacterial dominant phylum and diversity. Table S2. The topological properties of bacterial genus co‐occurrence networks. Table S3. Module scores and major taxa abundance in the hold three bacterial genus networks. Table S4. Importance ranking of factors that significantly affect genus structure. Table S5. The topological properties of the metabolic functional co‐occurrence networks on two KEGG ko levels. Table S6. Module scores and major taxa abundance in the functional differential networks. [file ECE3-15-e71096-s002.docx]

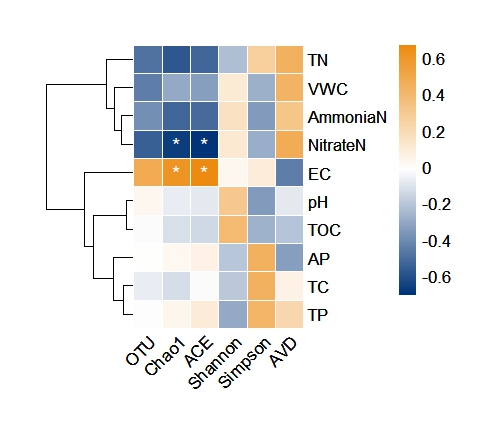


**Fig. S1** The effect of soil physicochemical properties on bacterial diversity and average variation degree. * indicate significant differences between soils in two sites, with *p* < 0.05.

**Table S1** The relation between bacterial dominant phylum and diversity

|  | AVD | chao | ace | shannon | simpson |
| --- | --- | --- | --- | --- | --- |
| Nitrospirae | 0.042 | -0.196 | -0.231 | 0.503 | -0.727** |
| Proteobacteria | 0.657* | -0.657* | -0.643* | -0.357 | 0.196 |
| Nitrospinae | 0.657* | -0.538 | -0.566 | -0.154 | 0.077 |
| Others (rare) | -0.413 | 0.308 | 0.245 | 0.818** | -.0825** |

**Table S2** The topological properties of bacterial genus co-occurrence networks

| Topological properties | Jiangsu | Fujian | Difference |
| --- | --- | --- | --- |
| Number of nodes | 152 | 151 | 143 |
| Number of links | 532 | 626 | 249 |
| Positive correlations | 86.28% | 78.59% | 83.13% |
| Negative correlations | 13.72% | 21.41% | 16.87% |
| Average connectivity (avgK) | 7 | 8.29 | 3.48 |
| Modularity | 0.54 | 0.49 | 0.71 |
| Average clustering coefficient (avgCC) | 0.43 | 0.46 | 0.35 |
| Average path distance (APD) | 3.56 | 3.18 | 5.04 |
| Graph density (GD) | 0.046 | 0.055 | 0.025 |

**Table S3** Module scores and major taxa abundance in the hold three bacterial genus networks

| Module scores | Jiangsu | Fujian | Differential network | Major taxa  abundance | Jiangsu | Fujian | | Differential network | |
| --- | --- | --- | --- | --- | --- | --- | --- | --- | --- |
| Module1 | 26.97% | 21.85% | 17.48% | Proteobacteria | 49.34% | 49.67% | 50.35% | |  |
| Module2 | 21.71% | 20.53% | 16.78% | Bacteroidota | 13.16% | 13.25% | 11.89% | |  |
| Module3 | 18.42% | 20.53% | 15.38% | Acidobacteriota | 9.21% | 9.27% | 9.79% | |  |
| Module4 | 13.16% | 15.23% | 10.49% | Firmicutes | 5.26% | 6.62% | 4.9% | |  |
| Module5 | 5.92% | 10.6% | 9.09% | Actinobacteriota | 3.95% | 3.31% | 4.2% | |  |
| Module6 | 2.63% | 3.97% | 6.29% | Verrucomicrobiota | 2.63% | 1.99% | 2.8% | |  |
| Module7 | 1.97% | 1.99% | 5.59% | Chlamydiae | 2.63% | 1.99% | 2.1% | |  |
| Module8 | 1.32% | 1.32% | 2.1% | Nitrospinae | 1.97% | 1.99% | 2.1% | |  |
| Module9 | 1.32% | 1.32% | 2.1% | Chloroflexi | 1.97% | 1.99% | 2.1% | |  |
| Module7 | 1.32% | 1.32% | 2.1% | Spirochaetes | 1.32% | 1.32% | 1.4% | |  |
| Module10 | 1.32% | 1.32% | 1.4% | Planctomycetota | 1.32% | 1.32% | 1.4% | |  |
| Remains | ≤ 1.32% | ≤ 1.32% | ≤ 1.4% | Remains | ≤ 0.66% | ≤ 0.66% | | ≤ 0.7% | |

**Table S4** Importance ranking of factors that significantly affect genus structure

| Sorting | Factors | F | Proportion |
| --- | --- | --- | --- |
| 1 | Proteobacteria-*Other* | 50.9 | 0.002 |
| 2 | Acidobacteria-*Gp17* | 46.2 | 0.002 |
| 3 | Acidobacteria-*Gp9* | 38.4 | 0.004 |
| 4 | Acidobacteria-*Gp10* | 31.1 | 0.018 |
| 5 | Nitrate-N | 19.8 | >0.05 |
| 6 | Proteobacteria- *Syntrophobacter* | 15.8 | >0.05 |
| 7 | Proteobacteria-*Coxiella* | 15.7 | >0.05 |
| 8 | EC | 14.8 | >0.05 |
| 9 | Chloroflexi-Other | 11.5 | >0.05 |
| 10 | Amini-*Aminicenantes* | 4.7 | >0.05 |
| 11 | Proteobacteria-*Anderseniella* | 4.2 | >0.05 |
| 12 | Proteobacteria-  *Pleomorphobacterium* | 1.7 | >0.05 |

**Table S5** The topological properties of the metabolic functional co-occurrence networks on two KEGG ko levels

| Topological properties | Function difference | |
| --- | --- | --- |
|  | KEGG ko level 2 | KEGG ko level 3 |
| Number of nodes | 23 | 141 |
| Number of links | 187 | 4373 |
| Positive correlations | 100% | 99.98% |
| Negative correlations | 0% | 0.02% |
| Average connectivity (avgK) | 16.261 | 62.028 |
| Average clustering coefficient (avgCC) | 0.896 | 0.814 |
| Average path distance (APD) | 1.273 | 1.758 |
| Modularity | 0.054 | 0.186 |
| Graph density (GD) | 0.297 | 0.297 |

**Table S6** Module scores and major taxa abundance in the functional differential networks

| Module scores | KO level 2 | KO level 3 | Major functional abundance | KO level 3 |
| --- | --- | --- | --- | --- |
| Module1 | 56.52% | 49.65% | Metabolism of cofactors  and vitamins | 12.06% |
| Module2 | 26.09% | 47.52% | Xenobiotics biodegradation  and metabolism | 12.77% |
| Module3 | 17.39% | 1.42% | Amino acid metabolism | 14.89% |
| Module4 | - | 1.42% | Lipid metabolism | 6.38% |
|  |  |  | Energy metabolism | 5.67% |
|  |  |  | Carbohydrate metabolism | 7.09% |
|  |  |  | Replication and repair | 4.96% |
|  |  |  | Metabolism of terpenoids  and polyketides | 4.26% |
|  |  |  | Folding, sorting and degradation | 1.42 |
